# Acceptance of COVID-19 vaccination among maintenance hemodialysis patients: an Egyptian survey study

**DOI:** 10.1186/s41182-022-00434-3

**Published:** 2022-06-30

**Authors:** Samar Tharwat, Marwa K. Khairallah, Mohammed Kamal Nassar, Dalia Kamal Nassar, Eman Nagy

**Affiliations:** 1 Rheumatology & Immunology Unit, Department of Internal Medicine, Faculty of Medicine, Mansoura University, Mansoura, Egypt; 2 Assiut Nephrology & Dialysis Unit, Department of Internal Medicine, Faculty of Medicine, Assiut University, Assiut, Egypt; 3 Mansoura Nephrology & Dialysis Unit (MNDU), Department of Internal Medicine, Faculty of Medicine, Mansoura University, Mansoura, Egypt; 4 Medical Microbiology and Immunology Department, Faculty of Medicine, Mansoura University, Mansoura, Egypt

**Keywords:** COVID-19, Vaccination, Hemodialysis, Hesitancy, Acceptance

## Abstract

**Background:**

Coronavirus disease 2019 (COVID-19) is a contagious disease that is associated with significant morbidity and mortality especially among maintenance hemodialysis (MHD) patients. COVID-19 vaccination is important to decrease risk and severity of COVID-19 infection. However, vaccine hesitancy is a significant barrier to vaccination. Thus, the aim of this study was to investigate the vaccine acceptability among Egyptian MHD patients.

**Methods:**

We conducted a paper-based survey on 237 MHD patients in 2 tertiary Egyptian hemodialysis (HD) centers. The survey consisted of a questionnaire that addressed demographic and clinical data, knowledge and attitudes towards COVID-19 infection and vaccines, beliefs regarding both conventional and COVID-19 vaccines, intention of COVID-19 vaccination together with motivators for and barriers against vaccination, sources of information regarding COVID-19 vaccines.

**Results:**

According to intention to be vaccinated, the patients were divided into vaccine acceptant (VA), hesitant (VH), and resistant (VR) groups who comprised 58.3%, 26.5%, and 15.2%, respectively. Only occupational status and residency were significantly different between the three groups. In 60% of VA group, fear of infection was the main motivator for vaccination. Meanwhile, 40% of VH and VR groups reported that fear of serious side effects of vaccines was the main barrier against vaccination. Television was the primary information source (58.6%) about COVID-19 vaccination while only 18% of patients got their COVID-19 vaccine information from their nephrologists.

**Conclusions:**

More than half of MHD patients accept to receive COVID-19 vaccine. Vaccine acceptability is not associated with age, gender, educational level, but rather with employment status and residency.

## Background

Coronavirus disease 2019 (COVID-19) infection is caused by severe acute respiratory syndrome coronavirus 2 (SARS-CoV-2) which is a single-stranded RNA virus. It was first detected in December 2019 in Wuhan, China, and has spread rapidly worldwide. It was declared a global pandemic on 11th March 2020 [1, 2]. The World Health Organization (WHO) claimed that there were approximately 179 million confirmed COVID-19 cases worldwide with 3,895,661 deaths by June 25, 2021. Of them, 279,184 have been confirmed in Egypt with 16,002 deaths [3].

Maintenance hemodialysis (MHD) patients constitute a high-risk group for getting COVID-19 infection with reported high mortality rates [4]. That is because of associated comorbidities like diabetes mellitus, hypertension, cardiovascular disease, older age as well as presence of uremia-induced impaired immune response and pro-inflammatory state [5].

For prevention or reducing the risk of infection with COVID-19, non-pharmacological interventions, like wearing facial masks, keeping social distance, frequent cleaning and applying isolation measures for COVID-19 patients, are effective but challenging to implement in clinical practice [6]. Thus, COVID-19 vaccines are considered the most promising method to decrease the risk of infection. Numerous vaccines had become available for use in different parts of the world by the end of 2020. To date, there are 128 vaccines in clinical trials and 194 ones in preclinical stages [7].

Vaccine hesitation is defined as "the delay in accepting or refusing vaccines despite the availability of the vaccination services [8]. It is a significant obstacle to reaching the requisite vaccination levels to contain the pandemic [9]. Given that one in three HD claimed prior personal contact with COVID-19 and one in four stated a close family member had antecedent infection, vaccine hesitancy in the high-risk dialysis population is alarming [10]. HD Patients have been reported to show high levels of vaccine hesitancy [11, 12]. Vaccine hesitancy among these patients is not linked to their educational level, age, or gender, but rather to a lack of confidence in vaccine efficacy and safety concerns [11]. Fortunately, the number of COVID-19 vaccination doses administered per 100 people in Egypt rose to 82 as of Jun 3, 2022 [13], however, data on HD patients are lacking.

To the best of our knowledge, only few studies have shown the pattern of COVID-19 vaccine acceptability among MHD patients. Understanding the characteristics that contribute to COVID-19 vaccine intention and behavior in MHD patients with possible reluctance or hesitancy of COVID-19 vaccines is critical for developing effective methods to enhance COVID-19 vaccination. This study aimed to establish the level of COVID-19 vaccine acceptance, hesitancy, and resistance among Egyptian MHD patients as well as the motivations and impediments that may influence their vaccination decision-making.

## Patients and methods

### Study design and settings

This observational cross-sectional survey study was conducted on MHD patients aged 18 years or more and followed up by staff members at 2 central Egyptian HD units: Dakahlia and Assiut university hospitals from 15th August to 5th September 2021. Patients with psychological or neurological disabilities that interfere with their response to the questionnaire were excluded from the study. This sample size calculation was conducted based on G*Power; the outcome of interest is COVID-19 vaccine acceptability rate. It was 72% [14], alpha error 0.05, and power of study 0.95. So, the sample size was found to be 192 subjects.

Convenience sampling technique was used for recruitment. The study was explained in detail to all participants. An informed written consent was obtained from participants before enrollment in the study. The work complies with the 1995 Helsinki Declaration’s ethical principles and has been authorized by the corresponding universities’ local institutional ethical committees (Approval No: R.21.09.1452). Personal meetings with the studied patients were carried out and the questionnaire was explained to each patient. Participants with a low education level were instructed on how to complete the questionnaire. The questionnaire was distributed among the participants by the researchers. It was administered in two ways: self-administered for individuals who could read and write, and interview questionnaire for those who could not.

### Questionnaire

Researchers designed and pretested the questionnaire after an extensive literature review [15, 16]. The first author created the questionnaire form, which was then modified by the other authors. Some modifications were performed to it to be suitable for MHD patients. Then, it was translated to Arabic and distributed in paper form to the patients. The developed questionnaire was subjected to evaluation by a team of 5 nephrology staff members for their inputs, critical appraisal, and content validation. On this basis, no new items were inserted, 5 items were deleted and 3 were reworded. Then a preliminary questionnaire was built up and pretested in a small group of MHD patients (*n* = 20). Cronbach's alpha coefficient was used to determine the questionnaire's internal consistency. The reliability coefficient was 0.87, indicating a good internal consistency. The questionnaire contained multiple choice questions and it was composed of 6 sections. The first section addressed demographic data of patients including age, sex, marital status, occupation, residency, educational level, smoking habit, lifestyle, and family income. Clinical data were evaluated by questions in the second section and included cause of chronic renal failure (CRF), HD duration, self-rated overall disease status, associated comorbidities, therapeutic history, drug adherence, and history of COVID-19 infection among patients and their acquaintances. The third section comprised questions about knowledge (assessed by a scale from very bad to very good), attitudes, perception about COVID-19 infection and vaccine as well as beliefs about conventional vaccination.

**Fig. 1:**
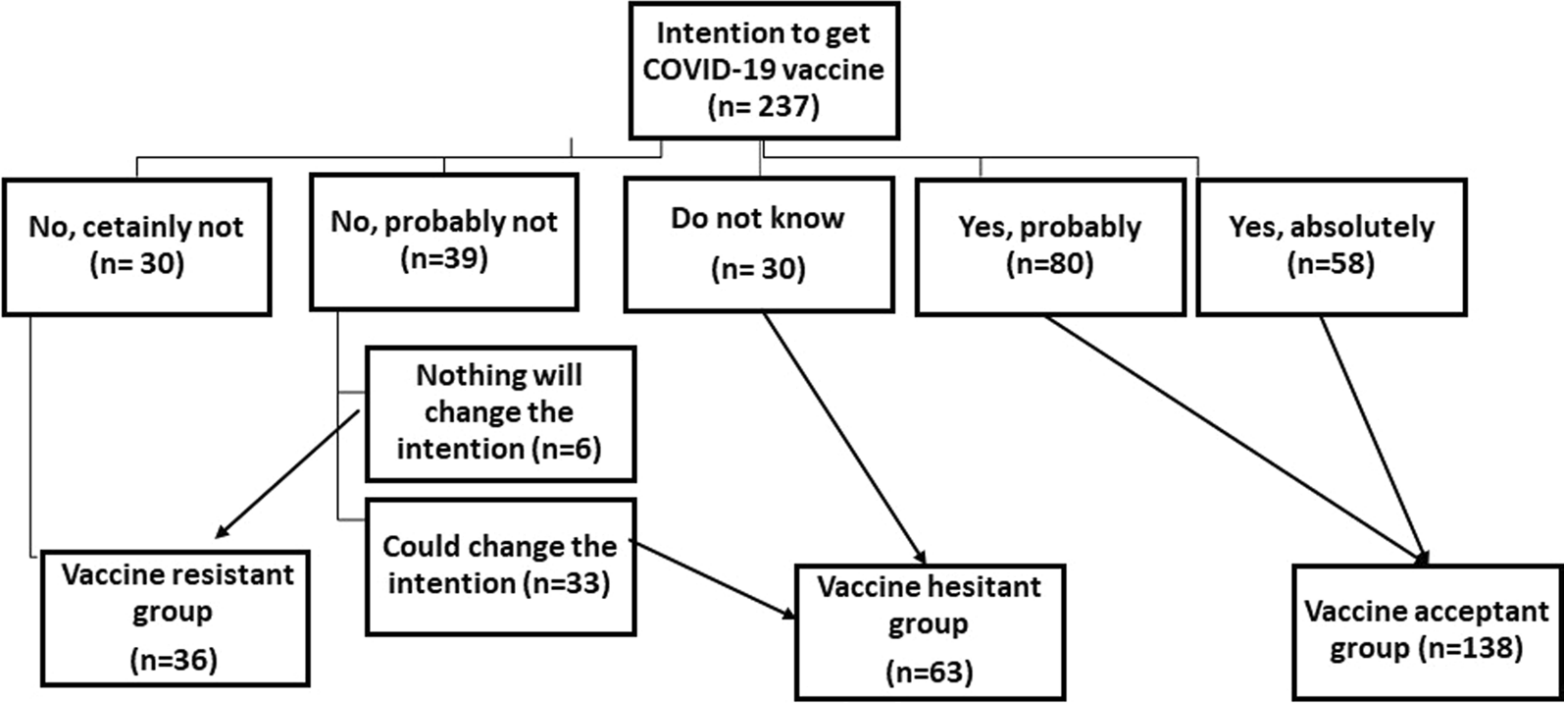
Classification of the participants according to intention to receive COVID-19 vaccine (resistant, hesitant, and acceptant)

The fourth section was about assessing the beliefs of MHD patients regarding COVID-19 vaccine. The fifth section addressed intention to get a COVID-19 vaccine by using five-point Likert scale question. Participants' vaccine acceptance was regarded as a primary endpoint. If they chose the options “Yes, absolutely” or “Yes, probably”, they were called vaccine acceptant (VA). They were labeled vaccine hesitant (VH) if they answered "No, probably not" or "I don't know" to the question about vaccine intention. Participants who replied "No, certainly not" or "No, probably not" and "Nothing will change the intention" were considered vaccine resistant (VR). The classification is depicted in the flowchart (Fig. 1).

The fifth section addressed motivators for and barriers against COVID‐19 vaccination and reasons that would change decision about getting COVID-19 vaccination among VH group. In addition, questions for patients’ sources of information about COVID-19 vaccine and their different types were included in this section.

The last section was related to actual uptake of COVID-19 vaccine. We asked about the type of the administered vaccine in vaccinated patients and the observed side effects, namely, fever, chills, anaphylaxis, widespread muscle or joint pain, headache, nausea, vomiting, skin rash, poor appetite, fatigue, sleepiness, chest pain or palpitations. Their responses were lastly transferred into an excel spreadsheet.

### Statistical analysis

The collected data were analyzed using the Statistical Package for Social Science (SPSS) version 25. Qualitative data were expressed as numbers and percentages, while quantitative data were described as means ± standard deviation (SD) for parametric variables or medians (min–max) for nonparametric variables. To assess the normality of distribution of variables, Shapiro–Wilk test were used. For comparing between the study groups, one way ANOVA test was used for parametric variables, while Kruskal–Wallis test was used for nonparametric variables. Chi-square test was used for comparing between qualitative variables. P value less than 0.05 was considered to be significant.

## Results

The current study initially included 250 MHD patients, however 13 were excluded due to missed or invalid data. Thus, 237 MHD patients constituted the study group. They had a mean age of 45.25 ± 18.27 years and 123 (51.9%) were males. Fifty percent of patients did not have jobs while 21.9% of patients were not educated. Sixty two percent lived in rural areas and 66.7% had active lifestyle. About 25% did not have enough income. Forty-four patients (18.6%) were infected with COVID-19 with a median duration of COVID-19 manifestations of 9.5 days. From these patients, 13 patients were hospitalized and only one patient needed intensive care unit (ICU) admission (Table 1).

**Table 1:** Demographic and clinical data of hemodialysis patients according to intention to get the COVID-19 vaccine

Variables	Total	VA	VH	VR	*P*
	(*n* = 237)	(*n* = 138)	(*n* = 63)	(*n* = 36)	
Age (years), mean ± SD	45.25 ± 18.27	46.77 ± 17.88	41.44 ± 17.32	46.08 ± 20.77	0.14
Sex					
Females	114 (48.1)	62 (44.9)	35 (55.6)	17 (47.2)	0.375
Males	123 (51.9)	76 (55.1)	28 (44.4)	19 (52.8)	
Marital status					
Single/divorced/widowed	52 (21.9)	31 (22.5)	14 (22.2)	7 (19.4)	0.925
Married	185 (78.1)	107 (77.5)	49 (77.8)	29 (80.6)	
Occupation					
Unemployed	119 (50.2)	61 (44.2)	35 (55.6)	23 (63.9)	0.018*
Employed	42 (17.7)	24 (17.4)	13 (20.6)	5 (13.9)	
Retired	28 (11.8)	16 (11.6)	7 (11.1)	5 (13.9)	
Cannot work due to disability	45 (19)	34 (24.6)	8 (12.7)	3 (8.3)	
Student	3 (1.3)	3 (2.2)	0	0	
Education level					
Not educated	52 (21.9)	34 (24.6)	7 (11.1)	11 (30.6)	0.215
Low school	30 (12.7)	17 (12.3)	7 (11.1)	6 (16.7)	
Middle school	90 (38.0)	43 (31.2)	35 (55.6)	12 (33.3)	
High school	2 (0.8)	1 (0.7)	1 (1.6)	6 (16.7)	
College degree	60 (25.3)	41 (29.7)	13 (20.6)	1 (2.8)	
Post-graduate	3 (1.3)	2 (1.4)			
Governorate					
Assiut	148 (62.4)	87 (63)	36 (57.1)	25 (69.4)	0.467
Dakahlia	89 (37.6)	51 (37)	27 (42.9)	11 (30.6)	
Residence					
Rural	149 (62.9)	79 (57.2)	40 (63.5)	30 (83.3)	0.016*
Urban	88 (37.1)	59 (42.8)	23 (36.5)	6 (16.7)	
Active lifestyle	158 (66.7)	92 (66.7)	42 (66.7)	24 (66.7)	1
Smoking habit					
Nonsmoker	193 (81.4)	111 (80.4)	55 (87.3)	27 (75)	0.277
Former smoker	25 (10.5)	15 (10.9)	5 (7.9)	5 (13.9)	
Current Smoker	19 (8)	12 (8.7)	3 (4.8)	4 (11.1)	
Family income					
Not enough	61 (25.7)	31 (22.5)	18 (28.6)	12 (33.3)	0.132
Enough with no saving	156 (65.8)	91 (65.9)	44 (69.8)	21 (58.3)	
Enough and saving	20 (8.4)	16 (11.6)	1 (1.6)	3 (8.3)	
Causes of chronic renal failure					
Amyloidosis	1 (0.4)	0	1 (1.6)	0	0.921
Analgesic nephropathy	10 (4.2)	4 (2.9)	5 (7.9)	1 (2.8)	
Chronic pyelonephritis	5 (2.1)	2 (1.4)	2 (3.2)	1 (2.8)	
Congenital kidney abnormalities	17 (7.2)	11 (8)	5 (7.9)	1 (2.8)	
Diabetes mellitus	9 (3.8)	2 (1.4)	5 (7.9)	2 (5.6)	
Glomerulonephritis	9 (3.8)	5 (3.6)	2 (3.2)	2 (5.6)	
Hypertension	65 (27.4)	45 (32.6)	12 (19)	8 (22.2)	
Hypertension and diabetes mellitus	18 (7.6)	12 (8.7)	4 (6.3)	2 (5.6)	
Hypovolemia	5 (2.1)	2 (1.4)	3 (4.8)	0	
Nephrolithiasis	6 (2.5)	4 (2.9)	1 (1.6)	1 (2.8)	
Pregnancy related	4 (1.7)	2 (1.4)	1 (1.6)	1 (2.8)	
Schistosomiasis	1 (0.4)	1 (0.7)	0	0	
Unknown	87 (36.7)	48 (34.8)	22 (34.9)	17 (47.2)	
Disease duration, months, median (min–max)	36 (1–288)	36 (1–288)	36 (1.2–204)	48 (1.2–192)	0.363
Self-rated overall disease activity, median (min–max)	5 (0–10)	5 (0–10)	5 (0–9)	5 (1–10)	0.599
Therapeutic data					
None	31 (13.1)	17 (12.3)	7 (11.1)	7 (19.4)	0.458
Erythropoietin	151 (63.7)	88 (63.8)	43 (68.3)	20 (55.6)	0.451
Iron supplementation	153 (64.6)	89 (64.5)	40 (63.5)	24 (66.7)	0.95
Calcium supplementation	162 (68.4)	97 (70.3)	43 (68.3)	22 (61.1)	0.575
Vitamin D	117 (49.4)	75 (54.3)	28 (44.4)	14 (38.9)	0.17
Calcimimetics	14 (5.9)	7 (5.1)	6 (9.5)	1 (2.8)	0.32
Aluminum hydroxide	5 (2.1)	2 (1.4)	1 (1.6)	2 (5.6)	0.296
Antihypertensives drugs	132 (55.7)	78 (56.5)	39 (61.9)	15 (41.7)	0.144
Antidiabetic drugs	33 (13.9)	19 (13.8)	7 (11.1)	7 (19.4)	0.515
Adherence to therapy	204 (86.1)	120 (87)	54 (85.7)	30 (83.3)	0.852
Associated comorbidities					
Diabetes	42 (17.7)	24 (17.4)	11 (17.5)	7 (19.4)	0.958
Hypertension	167 (70.5)	101 (73.2)	46 (73.0)	20 (55.6)	0.105
Chronic lung disease	5 (2.1)	4 (2.9)	1 (1.6)	0	0.53
Ischemic heart disease	13 (5.5)	8 (5.8)	3 (4.8)	2 (5.6)	0.956
COVID-19 infection					
History of COVID-19 infection	44 (18.6)	27 (19.6)	11 (17.5)	6 (16.7)	0.893
Duration, days, median (min–max)	9.5 (0–120)	9 (0–25)	14 (1–120)	4 (1–14)	445
Hospitalization	13 (29.6)	8 (29.6)	5 (45.5)	0	0.202
ICU admission	1 (2.3)	0	1 (9.5)	0	0.256
COVID-19 among relatives	65 (27.4)	41 (29.7)	12 (19)	12 (33.3)	0.202
Hospitalization	21 (8.9)	14 (10.1)	2 (3.2)	5 (13.9)	0.141
Death	20 (8.4)	13 (9.4)	3 (4.8)	4 (11.1)	0.449

^*^ VA: vaccine acceptant; VH: vaccine hesitant; VR: vaccine resistant, *P* value < 0.05

The patients were divided according to their intention to be vaccinated into 3 groups which were VA (138 patients, 58.3%), VH (63 patients, 26.5%), and VR (36 patients, 15.2%) (Fig. 1). The three groups were compared regarding different clinical and medical characteristics. Comparison of occupational status and residency between the three groups yielded statistically significant differences. However, no significant differences were detected between the three groups regarding age, sex, marital status, educational level, lifestyle, and family income (Table 1).

Regarding knowledge and perception about COVID-19 infection and vaccines, there was no statistical difference regarding knowledge among the three groups. Meanwhile, perception of having a higher risk to be infected with COVID-19, to develop more severe disease, and to develop adverse events after vaccination due to CRF, showed statistically significant differences between the three groups (Table 2).

**Table 2:** Knowledge, attitudes, perception about COVID-19 infection and vaccine among participants (*n* = 237)

Variables	Total	VA	VH	VR	*P*
	(*n* = 237)	(*n* = 138)	(*n* = 63)	(*n* = 36)	
Self-rated knowledge level about COVID-19					
Very bad	22 (9.3)	12 (8.7)	6 (9.5)	4 (11.1)	0.226
Bad	72 (30.4)	37 (26.8)	19 (30.2)	16 (44.4)	
Average	127 (53.6)	80 (58)	34 (54)	13 (36.1)	
Good	12 (5.1)	6 (4.3)	3 (4.8)	3 (8.3)	
Very good	4 (1.7)	3 (2.2)	1 (1.6)	0	
COVID-19 information sources					
Very little	11 (4.6)	2 (1.4)	6 (9.5)	3 (8.3)	0.165
Little	69 (29.1)	38 (27.5)	17 (27)	14 (38.9)	
Somewhat	133 (56.1)	84 (60.9)	35 (55.6)	14 (38.9)	
Much	21 (8.9)	11 (8)	5 (7.9)	5 (13.9)	
So much	3 (1.3)	3 (2.2)	0		
Belief of chronic renal failure association with higher risk of COVID-19	122 (51.5)	88 (63.8)	30 (47.6)	4 (11.1)	<0.001*
Perception of increased severity of COVID-19 in patients with chronic renal failure	122 (51.5)	84 (60.9)	32 (50.8)	6 (16.7)	<0.001*
Beliefs about conventional vaccination					
Efficacy, median (min–max)	5 (0–10)	6 (0–10)	5 (0–10)	5 (0–10)	<0.001*
Security, median (min–max)	5 (0–10)	6.5 (0–10)	5 (0–10)	5 (0–10)	<0.001*
Usefulness, median (min–max)	6 (0–10)	7 (0–10)	6 (0–10)	5 (0–10)	0.004*
Estimated knowledge, median (min–max)	5 (0–10)	5 (0–10)	5 (0–10)	5 (0–10)	0.050*

^*^ VA: vaccine acceptant; VH: vaccine hesitant; VR: vaccine resistant, *P* value < 0.05

On measuring self-estimated knowledge about conventional vaccines by a scale from 1 to 10, the median knowledge score was 5/10, while confidence in efficacy, safety and usefulness of vaccines were 5/10, 5/0, and 6/10, respectively. Comparing these results between the three groups showed statistically significant differences (Table 2). Beliefs of MHD patients regarding COVID‐19 vaccination among three groups are illustrated in Table 3.

**Table 3:** Beliefs of hemodialysis patients regarding COVID‐19 vaccination (*n* = 237)

Statement	Total (*n* = 237)	VA (*n* = 138)	VH (*n* = 63)	VR (*n* = 36)	*P*
COVID‐19 vaccine is important	153 (64.6)	119 (86.2)	28 (44.4)	6 (16.7)	<0.001*
COVID‐19 vaccination to everyone in the community is important	139 (58.6)	112 (81.2)	22 (34.9)	5 (13.9)	<0.001*
COVID‐19 vaccination should always be compulsory	136 (57.4)	108 (78.3)	22 (34.9)	6 (16.7)	<0.001*
Concerns about COVID‐19 vaccination	141 (59.5)	116 (84.1)	22 (34.9)	3 (8.3)	<0.001*
COVID‐19 vaccination of should always be compulsory for HCWs	166 (70)	119 (86.2)	32 (50.8)	15 (41.7)	<0.001*
Approval of the vaccine guarantees its safety	107 (45.1)	85 (61.6)	13 (20.6)	9 (25)	<0.001*
Vaccination is the best preventive measure for COVID‐19	124 (52.3)	102 (73.9)	18 (28.6)	4 (11.1)	<0.001*
COVID‐19 vaccine may have adverse effects	174 (73.4)	107 (77.5)	45 (71.4)	22 (61.1)	0.129
COVID‐19 vaccine may be ineffective	165 (69.6)	101 (73.2)	45 (71.4)	19 (52.8)	0.057
A prior bad experience with any vaccines	30 (12.7)	22 (15.9)	5 (7.9)	3 (8.3)	0.201
Against vaccination in general	33 (13.9)	11 (8)	15 (23.8)	7 (19.4)	0.006*
I fear getting COVID‐19 infection from the vaccine	152 (64.1)	89 (64.5)	43 (68.3)	20 (55.6)	0.445
You are not at elevated risk of complications following COVID‐19 infection	98 (41.4)	54 (39.1)	29 (46)	15 (41.7)	0.655
You are not at high risk to get COVID‐19 infection	117 (49.4)	69 (50)	33 (52.4)	15 (41.7)	0.577

^*^ VA: vaccine acceptant; VH: vaccine hesitant; VR: vaccine resistant, *P* value < 0.05

**Fig. 2:**
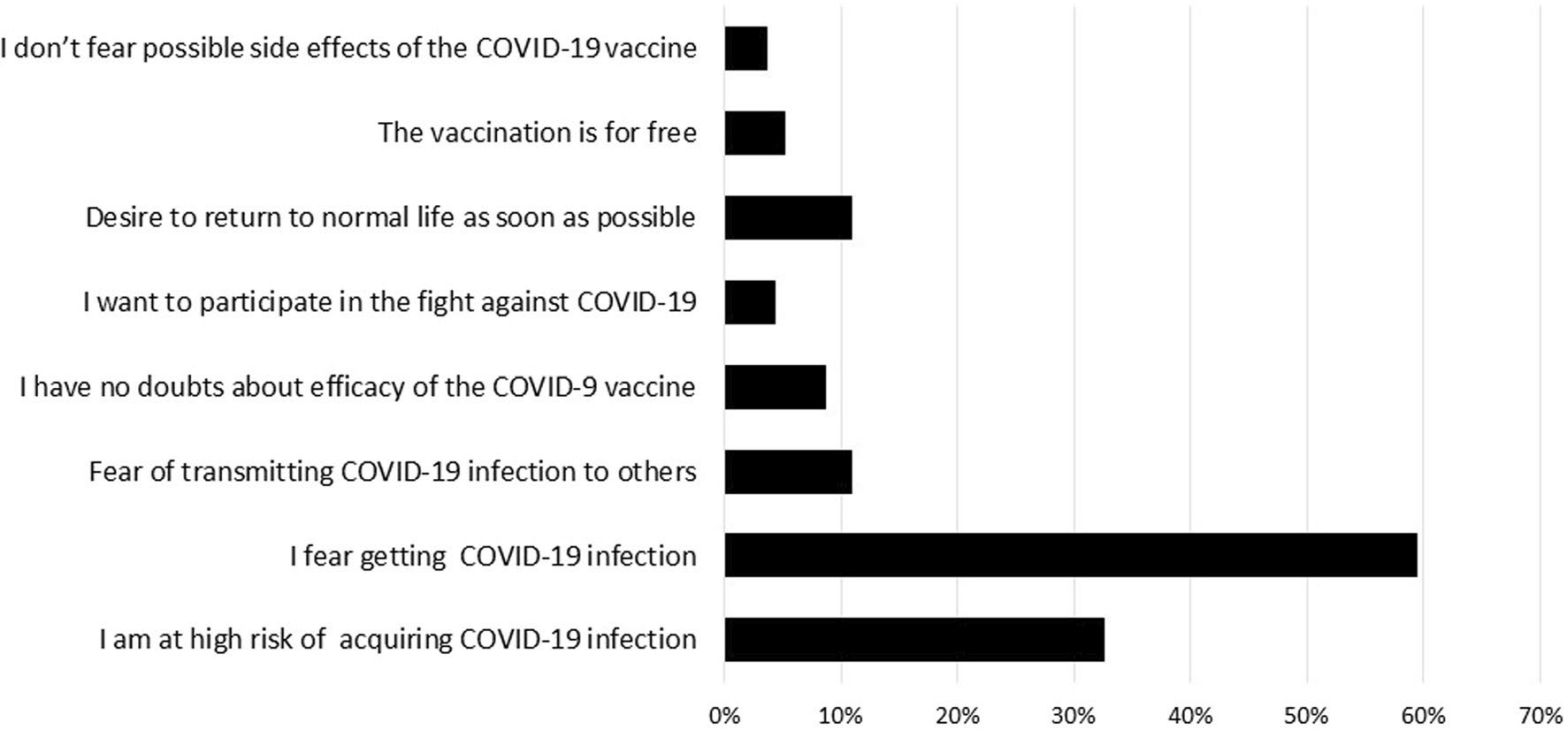
The motivators for COVID‐19 vaccination among the vaccine acceptant group of hemodialysis patients [*n* = 138]

**Fig. 3:**
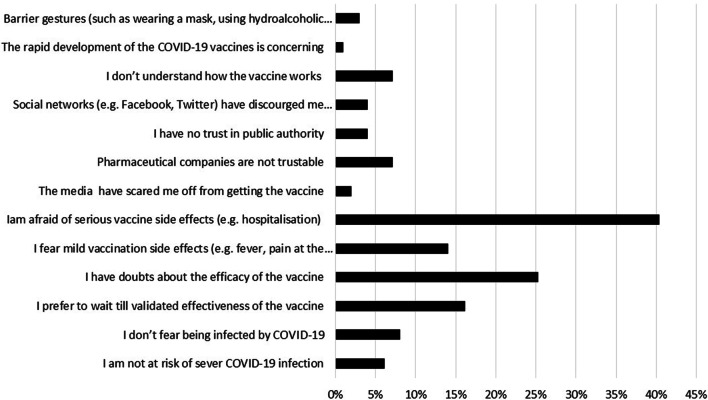
Hemodialysis patients’ barriers explaining COVID-19 vaccine hesitancy or resistance [*n* = 99]

**Fig. 4:**
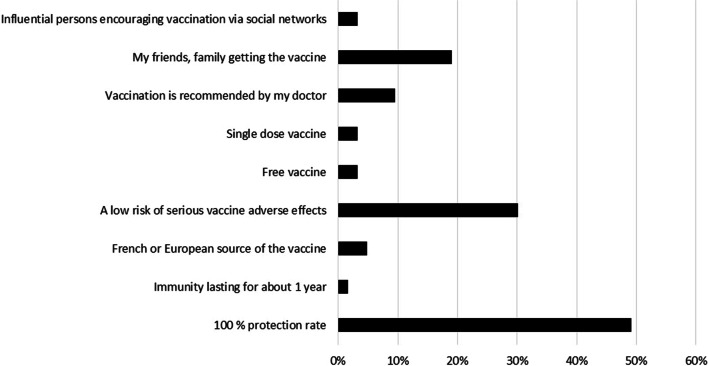
Reasons for change towards intention to get COVID-19 vaccination among vaccine hesitant group [*n* = 63]

Among VA group, “I do not want to be infected” was the most common motivator of COVID-19 vaccination followed by “I am a risk of COVID-19 infection” (59.4% and 32.6%, respectively) (Fig. 2). Meanwhile, the most common barriers against COVID-19 vaccination among VR and VH groups were “I fear of serious adverse effects of vaccine” followed by “I doubt the efficacy of vaccine” (40% and 25%, respectively) (Fig. 3). “A protection rate of 100%” followed by “a low risk of serious side effects” were the most chosen options that would change the intention of VH group towards getting a COVID-19 vaccine (49% and 30%, respectively) (Fig. 4).

Television followed by family and friends were reported by participants as their main information sources about COVID-19 vaccine (58.6%, 31.6% and 26.6%, respectively). On the contrary, newspapers, internet and general practitioner were the least sources among all the studied patients (3%, 8.9% and 10.5%, respectively). Eighteen percent of the studied patients got their information about COVID-19 vaccines from their nephrologists. When we asked the patients about the different types of COVID-19 vaccines, we found that 77% did not know the differences between them.

Thirty-six patients (15.2%) received COVID-19 vaccine. Astrazeneca and Sinopharm were the most common received vaccines (44.4% and 27.8%, respectively), while Pfizer and Sinovac were the least received vaccines (2.8% and 5.6%, respectively). Forty-seven percent of patients reported no adverse effects after vaccination. On the other hand, 33.3%, 27.8%, 13.9% complained of fever and/or chills, widespread muscle/joint pain, and fatigue/sleepiness, respectively. In addition, minority of patients complained of headache (8.3%), poor appetite (8.3%), vomiting (5.6%), nausea (2.8%), chest pain (2.8%) and rash (2.8%).

## Discussion

Maintenance HD patients are more exposed to infection with COVID-19 owing to comorbidities, immune dysfunction, in-center HD, and contact with other patients and medical staff [17]. Thus, COVID-19 vaccination together with social distancing and personal hygiene is important for reducing risk and severity of COVID-19 infection [18]. One of hurdles encountering mass vaccination against COVID-19 is vaccine hesitancy and resistance [19, 20].

In the current study, COVID-19 vaccine acceptability was identified in 58.3% in the studied MHD patients, while hesitancy and resistance were identified in 26.5% and 15.2% of them, respectively. Occupational status and residence of the patients were statistically different between VA, VH and VR groups. Fear of being infected was the common motivator to be vaccinated while fear of serious side effects was the main barrier to vaccination. Patients' knowledge about COVID-19 vaccines did not affect their response to vaccination.

Percentage of vaccine acceptability in the current study was lower than reported by Garcia et al. [14] who conducted an online survey study on 1515 patients in 150 randomly selected HD centers in the United States and found that about 80% of the patients were in favor of getting COVID-19 vaccine which is the same result of Andrian et al. [21], whose study consisted of a paper-based survey of 159 MHD French patients. Our results are also lower than that stated by Arce et al. [22] who investigated COVID-19 vaccine acceptance through a survey of general population in ten low- and middle-income countries and found that the acceptance levels ranged from 67 to 97%. However, our reported results are higher than those reported by Rungkitwattanakul et al. study [12], which was a paper-based survey study on 90 African American MHD patients and they found that 49% of the patients would be willing to receive the vaccine. These differences may be due to change in geographic areas, level of preexisting vaccine hesitancy, different interaction with healthcare providers, and quality of perceived information about COVID-19 vaccines.

Among demographic data of patients in the current study, occupational status and residence affected their response to vaccination, which means that unemployed patients and/or rural patients were significantly higher in the VH and VR groups. Age, gender, educational level did not affect the response of our patients to vaccination. Although Garcia et al. found that age, gender, and educational level affect the intention of COVID-19 vaccination in an online survey conducted on MHD patients [14], our study did not confirm these findings. In another survey conducted on MHD patients, Blanchi et al. stated that younger age was associated with vaccine hesitancy while hesitancy was not related to educational level and gender [11].

Around fifth of the studied patients had a history of COVID-19 infection in the previous 18 months which is close to the findings reported by Creput et al. [23] and Xiong et al. [24] studies. However, this result is higher than those stated by Yau et al. [25] and Quintaliani et al. [26] studies. Our reported result about incidence of COVID-19 among MHD patients is lower than that reported by La Milia et al. [27] study. About 30% of our COVID-19 infected patients needed hospitalization. This result is lower than stated by Yau et al. [25], Creput et al. [23] and Alberici et al. [28] studies. In the current study, only one patient (2.3%) needed ICU admission during COVID-19 infection, a result which is much lower than those found in Ma et al. [29] and Yau et al. [25] studies. These differences may be attributed to the fact that our study included patients who survived COVID-19 infection in contrast to other studies in which patients were followed up across the course of the disease.

In the present study, around half of the patients thought they were at a higher risk of contracting COVID-19 infection as well as being more prone to increased severity of infection due to CRF. These may be the causes behind vaccine acceptability among the studied patients as we observed that majority of VA patients had high levels of perception about risks and dangers of COVID-19 infection. On the other hand, just less than half of the studied patients believed they were at higher risk of COVID-19 vaccine adverse events due to CRF. This led to vaccine hesitancy among the patients.

Unexpectedly, level of knowledge and information sources about COVID-19 did not affect intention of the patients to be vaccinated in the current study. This result is in accordance with that reported by Blanchi et al., whose study conducted a survey with an ad hoc questionnaire on 417 MHD patients in 4 large HD centers in France and Italy [11] and Kerr et al., a study carried out on general population [30]. However, a nationwide survey in United States found that general vaccine knowledge was a significant predictor of COVID-19 vaccination intent [31].

Generally, conventional vaccines beliefs and behavior of people affect their knowledge and attitudes towards vaccination. Additionally, fears about vaccine safety, effectiveness, side effects and lack of knowledge are previously identified barriers to vaccination [32, 33]. Thus, the VA group in the current study had significantly more positive beliefs and attitudes, compared to VH and VR groups of patients, about conventional vaccine efficacy, security, and usefulness.

In the current study, the most common reported motivators of COVID-19 vaccination were fear of being infected and having high risk of infection, while small percentages of the patients selected avoiding transmission of disease to others, free vaccine, not worrying about the side effects, and trusting the efficacy of vaccine as motivators for COVID-19 vaccination. There are several motivators of COVID-19 vaccination among individuals in different studies. However, fear of infection remains a common factor among these studies [22, 34, 35]. A similar conclusion was reached by another study conducted on Egyptian healthcare workers, in which being at high risk of COVID-19 infection, trusting safety and effectiveness of vaccine and facilitation of travel were the main motivators of vaccination [34]. A similar pattern of results was also obtained in a survey study covering 10 low- and middle-income countries and revealed that desire for personal and family protection against infection were the common causes of vaccine acceptability [22]. In another study carried out on patients with cancer breast, the main motivators of vaccination were prevention of COVID-19 infection, protection of their families, being socially responsible, fear of getting extremely ill, and a desire for “getting back to normal” [35].

Generally, trust in vaccines and related administrative authorities are the main factors that determine success of any vaccination campaigns [36]. In the present study, there were numerous barriers to COVID-19 vaccination leading to vaccine hesitancy. Fear of serious side effects, doubt about the efficacy of vaccine and wishing to wait for more experience with the new vaccines were the main causes of vaccine hesitancy and resistance in the current study. These causes are in accordance with those reported by Ruiz and Bell in their nationwide survey of 804 U.S. adults [31]. The main causes of vaccine hesitancy among employees in a tertiary care hospital were doubts about COVID-19 vaccine safety and efficacy, fear of side effects, and having a history of previous COVID-19 infection [37]. Fear of adverse events and skepticism of the health care system were the common barriers to vaccination among patients with breast cancer [35].

Television was the main source of information regarding COVID-19 vaccine among the studied patients, followed by family, friends and social media. In an online survey on MHD patients in the United States, television followed by dialysis staff were the most common information sources about COVID-19 vaccine [14]. Meanwhile, an online survey study on 1011 Italian citizens conducted by Reno et al., television was the most used source to get information about COVID-19 vaccines followed by newspapers and institutional websites [38]. The source of information may increase or decrease the vaccine hesitancy. As an example of source of information that may mediate vaccine hesitancy is media focusing mainly on adverse events of vaccine, such as information about rare cases of thrombosis associated with the AstraZeneca vaccine, which may exacerbate concerns about side effects [39]. Although healthcare workers are trusted sources of information and can influence choice of the patients, only 18% of our patients got their information from nephrologists.

In the current study, around three quarters of patients did not know the differences between COVID-19 vaccine types. This result indicates lack of reliable information and knowledge about COVID-19 vaccines as they acquired this information mainly from social media. Thus, nephrologists and HD nurses should carry out awareness campaigns for MHD patients to enhance their knowledge about COVID-19 vaccines.

Gaining trust in a COVID-19 vaccine is important especially among high-risk population such as HD patients. We found that 55% of our study sample would accept COVID-19 vaccination. However, only 15% received the vaccine which is a much lower percentage than those who claimed the intended to be vaccinated. So, health care providers should encourage trust in COVID-19 vaccination and minimize misinformation. To combat misinformation and promote trust, deliberate and focused communications must be designed and tested now in order to increase current public interest. Messaging and education should not only be directed at the general population, but also at high-risk populations including MHD patients.

Being the first study of COVID-19 vaccine acceptability and hesitancy in Egyptian MHD patients besides using interview-based questionnaire are among the strengths of this study.

However, a number of potential limitations need to be considered. First, it was a convenience sample from different HD centers in Egypt; voluntary participation could have skewed the results and led to self-selection bias. Second, the cross-sectional nature of this study should not be ignored, as vaccine hesitancy is context-dependent, particularly in terms of the location and timing of any survey. Third, the dependence on self-reported data of the patients. Furthermore, because of the descriptive character of this study, it is possible to find only relationships between the studied variables. As a result, more research with a more robust experimental design is needed to evaluate the psychological drivers of COVID-19 vaccine hesitancy.

## Conclusions

More than half of MHD patients accept COVID-19 vaccination. Vaccine acceptability in MHD patients is not affected by age, gender, educational level, but rather influenced by occupational status and life in urban areas. Fear of getting infected by SARS-COV-2 is the main motivator for vaccination while fear of vaccine side effects is the main barrier against vaccine uptake. Television represents the main information source about COVID-19 vaccines, while nephrologists are unexpectedly a minor source of information for Egyptian MHD patients.

## Data Availability

The datasets used and/or analyzed during the current study are available from the corresponding author on reasonable request.

## Abbreviations

COVID-19: Coronavirus disease 2019
CRF: Chronic renal failure
HD: Hemodialysis
ICU: Intensive care unit
MHD: Maintenance hemodialysis
SAGE: Scientific Advisory Groups of Experts
SARS-CoV-2: Severe acute respiratory syndrome coronavirus 2
SD: Standard deviation
SPSS: Statistical Package for Social Science
VA: Vaccine acceptant
VH: Vaccine hesitant
VR: Vaccine resistant
WHO: World Health Organization
